# SARS-CoV and SARS-CoV-2 display limited neuronal infection and lack the ability to transmit within synaptically connected axons in stem cell–derived human neurons

**DOI:** 10.1007/s13365-023-01187-3

**Published:** 2024-01-03

**Authors:** Jasmina M. Luczo, Sarah J. Edwards, Katie Ardipradja, Willy W. Suen, Gough G. Au, Glenn A. Marsh, Nathan Godde, Christina L. Rootes, John Bingham, Vinod Sundaramoorthy

**Affiliations:** 1 Diagnostics, Surveillance and Response, Australian Centre for Disease Preparedness, Commonwealth Scientific and Industrial Research Organisation, Geelong, VIC Australia; 2 Health and Biosecurity, Australian Centre for Disease Preparedness, Commonwealth Scientific and Industrial Research Organisation, Geelong, VIC Australia; 3 School of Medicine, Deakin University, Geelong, VIC Australia

**Keywords:** Sarbecovirus, SARS, SARS-CoV-2, Neurotropism, Stem cell–derived neurons, Axonal trafficking, Trans-synaptic transmission

## Abstract

Sarbecoviruses such as SARS and SARS-CoV-2 have been responsible for two major outbreaks in humans, the latter resulting in a global pandemic. While sarbecoviruses primarily cause an acute respiratory infection, they have been shown to infect the nervous system. However, mechanisms of sarbecovirus neuroinvasion and neuropathogenesis remain unclear. In this study, we examined the infectivity and trans-synaptic transmission potential of the sarbecoviruses SARS and SARS-CoV-2 in human stem cell–derived neural model systems. We demonstrated limited ability of sarbecoviruses to infect and replicate in human stem cell–derived neurons. Furthermore, we demonstrated an inability of sarbecoviruses to transmit between synaptically connected human stem cell–derived neurons. Finally, we determined an absence of SARS-CoV-2 infection in olfactory neurons in experimentally infected ferrets. Collectively, this study indicates that sarbecoviruses exhibit low potential to infect human stem cell–derived neurons, lack an ability to infect ferret olfactory neurons, and lack an inbuilt molecular mechanism to utilise retrograde axonal trafficking and trans-synaptic transmission to spread within the human nervous system.

**Supplementary Information:**

The online version contains supplementary material available at 10.1007/s13365-023-01187-3.

## Background

The COVID-19 pandemic caused by severe acute respiratory syndrome coronavirus 2 (SARS-CoV-2) (genus *Betacoronavirus*, subgenus *Sarbecovirus*) has infected millions of people worldwide. The long-term human health impact of this pandemic is yet to be fully understood. There is now extensive evidence for acute and post-acute neurological symptoms from COVID-19. Acute COVID-19 neurological symptoms include myopathy, headache, encephalopathy, loss of smell or taste, delirium, seizures, and confusion (Rogers et al. 2021; Garcia-Monco et al. 2021; Misra et al. 2021; Romero-Sanchez et al. 2020; Flores-Silva et al. 2021; Douaud et al. 2022). However, people who have recovered from COVID-19 with initial mild symptoms can develop post-acute neurological sequela such as stroke, cognition and memory deficits, musculoskeletal disorders, mental health and psychiatric disorders (including psychosis), and peripheral nervous system disorders (Xu et al. 2022; Varatharaj et al. 2020; Nolen et al. 2022; Tang et al. 2022). A longitudinal study has further reported changes in brain structure following SARS-CoV-2 infection (Douaud et al. 2022). These neurological symptoms contribute to post COVID-19 chronic conditions collectively termed as long COVID. Furthermore, whether these neurological complications could increase risk of chronic neurological illness or neurodegenerative diseases in COVID-19 patients remains to be investigated. The cellular mechanisms contributing to COVID-19 neurological complications are still unclear, and there is limited evidence for SARS-CoV-2 central nervous system (CNS) invasion and direct neuronal cytopathology (Khan et al. 2021; Bauer et al. 2021; Butowt and von Bartheld 2022). Other sarbecoviruses such as severe acute respiratory syndrome coronavirus (SARS) and Middle East respiratory syndrome have also been associated with neurological complications (Kwong et al. 2020; Netland et al. 2008; Algahtani et al. 2016; Kim et al. 2017). Hence, it is essential to understand the potential mechanisms of sarbecovirus entry into the brain and the cause for associated neurological complications.

The brain is usually well protected from the entry of pathogens such as viruses by biological barriers including the blood–brain barrier; however, some neuroinvasive viruses have developed specific axonal trafficking and trans-synaptic transmission mechanisms to travel within the nerves and invade the brain. Since the onset of the COVID-19 pandemic, it has been widely suggested that the neurological symptoms observed could be caused by the virus invading the brain from the olfactory epithelium (Jiao et al. 2021; Rutkai et al. 2022; Meinhardt et al. 2021). The olfactory and other cranial nerves directly connect the olfactory epithelium with the olfactory bulb and the brain (Butowt and von Bartheld 2022; Sonne and Lopez-Ojeda 2022) which provides a direct anatomical route for SARS-CoV-2 to invade the brain from the nose. Such a potential mechanism has also been suggested during the 2003 SARS outbreak (Netland et al. 2008). Another human coronavirus, HC043, has been shown to enter the brain via the olfactory epithelium and cause encephalitis in mouse models (Netland et al. 2008; Desforges et al. 2019). To enable transmission within the nervous system via axons, viruses are known to interact and repurpose axonal trafficking machinery such as cytoskeletal proteins, molecular motors, and cargo adaptor molecules which are commonly present in all neuronal subtypes (Taylor and Enquist 2015). It is unknown whether sarbecoviruses have such an inherent ability to hijack axonal trafficking machinery and transmit between interconnected human neurons. Without an appropriate in vitro model, such an examination currently relies on analysing post-mortem tissues along the olfactory route and a correlation of viral positivity with the possible route taken by the virus.

To date, several studies have shown a potential for direct infection of SARS-CoV-2 in human brain neural cells including neurons both in in vitro neuronal models (Bauer et al. 2021; Zhang et al. 2020; McMahon et al. 2021; Ramani et al. 2020; Lyoo et al. 2022; Crunfli et al. 2022; Pepe et al. 2022; Kong et al. 2022) as well as following naturally acquired human infections and experimentally infected animals (Rutkai et al. 2022; Meinhardt et al. 2021; Bauer et al. 2022a; Beckman et al. 2022; Sia et al. 2020; de Melo et al. 2021; Kaufer et al. 2022; Stein et al. 2022). However, the efficiency of SARS-CoV-2 virus infection and replication in neurons is still unclear. While some initial studies reported a high degree of infection (Zhang et al. 2020; Ramani et al. 2020; Lyoo et al. 2022), others have reported limited ability of SARS-CoV-2 to infect and replicate in stem cell–derived human neuronal models (Bauer et al. 2021, 2022b). Studies have also identified higher infection rate of SARS-CoV-2 in non-neuronal CNS cells such as astrocytes (Kong et al. 2022; Andrews et al. 2021), microglia (Jeong et al. 2022; Albornoz et al. 2022), and choroid plexus epithelial cells (Jacob et al. 2020; Pellegrini et al. 2020), suggesting that replication in the brain could occur in non-neuronal cells and contribute to neuropathology. In post-mortem human brain tissues, studies have identified viral RNA and protein associated with neurons (Meinhardt et al. 2021; Stein et al. 2022; Matschke et al. 2020; Song et al. 2021). In addition, a recent study has isolated infectious virus from the thalamus and hypothalamus of one patient (Stein et al. 2022). However, other studies have reported exclusion of neuronal infection in COVID-19 patients (Khan et al. 2021; Butowt and von Bartheld 2022). It is unknown, whether this variability could be due to differences in viral probing techniques, individual patient heterogeneity, or stages of viral infection. The infectivity of SARS-CoV-2 in stem cell–derived neural models also appears to differ depending on whether culture system is a monolayer of co-cultured neural cells (Bauer et al. 2021, 2022b) or a complex three-dimensional (3D) organoid system (Zhang et al. 2020; Ramani et al. 2020; Mesci et al. 2022) composed of multiple neural cell types and layers. Another factor to be considered is whether the maturity of neurons during stem cell differentiation could influence the levels of SARS-CoV-2 infection and replication.

In this study, we used induced pluripotent stem cell (iPSC)–derived human forebrain neural cultures consisting of neurons and astrocytes to examine the infection and replication of the sarbecoviruses, SARS (Hong Kong strain), and SARS-CoV-2 ancestral and delta strains. Infection and replication of these sarbecoviruses were compared in stem cell–derived neural precursor cells (NPCs) as well as human neurons differentiated over medium (20–24 days) and long (50 days) terms. In this study, we observed infection of a small subset of neurons without productive replication over time (up to 72 h). In addition, for the first time, we tested the ability of SARS and SARS-CoV-2 virus to transfer between interconnected human neurons. We utilised our recently developed ex vivo model mimicking human neural network in a microfluidics device to examine the trans-synaptic transmission of sarbecoviruses. In this examination, we identified that both SARS and SARS-CoV-2 strains lack an inherent ability to transmit between human neurons within neurites. We then used SARS-CoV-2-infected ferret nasal turbinate tissues (Au et al. 2022) to examine SARS-CoV-2 infection of olfactory neurons. Consistent with our human ex vivo models, there was no detectable infection of olfactory neurons but a likely infection of sustentacular cells in the nasal epithelium similar to a previous finding in human tissues (Khan et al. 2021). Hence, our study adds further evidence for low potential of SARS and SARS-CoV-2 sarbecoviruses to infect and replicate in neurons. In addition, we demonstrate that these sarbecovirus strains do not possess an inherent ability to trans-synaptically transmit between interconnected human neurons in vitro. This supports the notion that sarbecoviruses do not cause widespread nervous system infection in humans and that neurologic complications could be consequences of immunopathology downstream to infection of non-neuronal cells such as astrocytes, or other pathology involving non-neural components such as blood vessels. Our findings also warrant investigation of alternate mechanisms of sarbecovirus entry into brain and the cause for observed neurological abnormalities independent of direct neuropathology.

## Methods

### Continuous cell lines

VERO C1008 (Vero E6) cells (ATCC CRL-1586™) were maintained in Dulbecco’s Modified Eagle Medium, high glucose, pyruvate, L-glutamine (Gibco) supplemented with 10% foetal bovine serum (CellSera), 10 mM HEPES (MP Biomedicals), and 1 × penicillin–streptomycin (Gibco) at 37 °C and 5% CO_2_.

BHK-21 cells (ATCC CCL-10™) were maintained in Minimum Essential Media supplemented with 10% foetal bovine serum, 10% tryptose phosphate broth, 10 mM HEPES, 2 mM glutamine (Gibco), and 1 × penicillin–streptomycin at 37 °C and 5% CO_2_.

### Stem cell–derived human neural cultures

The HDF51i-509iPSCs were differentiated into NPCs as described previously (Murphy et al. 2017). NPCs were then maintained in STEMdiff™ Neural Progenitor Medium (STEMCELL Technologies) supplemented with 1 × STEMdiff neural progenitor supplement A (STEMCELL Technologies) and 1 × STEMdiff neural progenitor supplement B (STEMCELL Technologies) at 37 °C and 5% CO_2_. Neural differentiation was initiated by replacing 50% v/v of the culture medium every 2–3 days with BrainPhysTM neural medium (STEMCELL Technologies) supplemented with 2% v/v Neurocult™ SM1 Neuronal Supplement (STEMCELL Technologies), 1% v/v N2 Supplement-A (STEMCELL Technologies), 20 ng/mL human recombinant brain-derived neurotrophic factor, 20 ng/mL human recombinant glial cell line–derived neurotrophic factor (Peprotech), 1 mM dibutyryl cyclic-AMP sodium salt (Sigma-Aldrich), and 200 nM ascorbic acid (STEMCELL Technologies), making complete BrainPhys™ neural medium (Sundaramoorthy et al. 2020a).

### Viruses

Severe acute respiratory syndrome coronavirus, HKU-39849 isolate (SARS-HK) (NCBI accession: AY278491.2), and SARS-CoV-2 SARS-CoV-2/human/AUS/VIC01/2020 (VIC01) (NCBI accession: MT007544.1), SARS-CoV-2/human/AUS/VIC02/2020 (VIC02) (NCBI accession: MT450919.1), and B.1.617.2 variant SARS-CoV-2/human/AUS/18440/2021 (Delta) strains were propagated in VERO C1008 cells, harvested by freeze–thaw ≤ 3 days post-inoculation, clarified by centrifugation, and stored at − 80 °C. Sarbecovirus titres were quantitated by titration in VERO C1008 cells. Infectious tissue culture supernatants were titrated tenfold and added to Vero C1008 monolayers in quadruplicate. Plates were incubated at 37 °C and 5% CO_2_ for 5 days, and cytopathic effect was visualised by light microscopy.

Rabies virus (RABV) CVS-11 strain was propagated in BHK-21 cells, harvested by freeze–thaw 5 days post-inoculation, clarified by centrifugation, and stored at − 80 °C. Virus titre was quantitated by titration in BHK-21 cells. Briefly, infectious tissue culture supernatants were titrated tenfold and added to BHK-21 monolayers in quadruplicate and incubated for 5 days at 37 °C and 5% CO_2_. RABV-infected monolayers were fixed with 4% paraformaldehyde (Sigma) in phosphate-buffered saline (PBS) (Thermo Fisher Scientific) at room temperature for ≥ 1 h or 4 °C overnight. Fixed monolayers were washed twice with PBS and FITC-conjugated anti-RABV monoclonal antibody cocktail (1:200) (Fujirebio Diagnostics) in PBS supplemented with 0.005% Evan’s Blue (Sigma), and 1% bovine serum albumin was added, and monolayers were incubated at 37 °C for 30 min. Monolayers were washed three times with PBS before adding PBS to each well. Fluorescence was visualised by fluorescent microscopy.

Virus stock titres were calculated by Reed and Muench method (Reed and Muench 1938). All work with infectious sarbecoviruses was performed at PC4/BSL4. All work with inactivated sarbecoviruses and RABV was performed at PC3/BSL3.

### Sarbecovirus infection of human iPSC–derived NPCs and neural cultures

HDF51i-509 NPCs (passage 11) were seeded at a density of 8 × 10^4^ cells/well in complete Neural Progenitor Medium in 24-well plates coated with 15 µg/mL poly-L-ornithine and 10 µg/mL laminin with or without glass coverslips (13 mm; Menzel Gläser). Undifferentiated NPCs were infected 24 h after seeding, or NPCs were differentiated for up to 50 days before infection. Neural cultures were infected at a multiplicity of infection (MOI) of 1 for up to 72 h. Briefly, appropriate volume of viral inoculum to infect at MOI 1 for each well was prepared in a total of 150 µL of BrainPhys™ neural medium. After removing the existing culture media in each well, 150 µL of diluted viral inoculum was added to the well, incubated at 37 °C and 5% CO_2_ for 30 min, and then, additional 500 µL of BrainPhys™ neural medium was added. Infected cultures were incubated at 37 °C and 5% CO_2_ for up to 72 h.

### Ex vivo model of interconnected human neurons using microfluidics

Xona microfluidic devices (Xona Microfluidics, Cat#SND450) were sterilised and plasma bonded to glass coverslips (24 × 40 mm; Menzel Glaser) using a plasma cleaner (PDC-32G-2, Harrick Plasma). After bonding, the devices were coated with 15 µg/mL poly-L-ornithine (Sigma) and 10 µg/mL laminin (Sigma). HDF51i-509 NPCs were seeded in both panels of a microfluidic device at a density of 8 × 10^4^ cells per panel in BrainPhys™ complete neural differentiation media to initiate differentiation. The media in the wells were replenished every 2–3 days and the differentiation was continued for up to 21 days.

Microfluidic chambers were infected as described previously (Sundaramoorthy et al. 2020a) with sarbecoviruses or RABV at a MOI 1 at day 20–21 of differentiation. Briefly, media from the panel to be infected was removed and the appropriate volume of viral inoculum in BrainPhys™ neural medium required to infect at MOI 1 was added. A unidirectional flow of media was strictly maintained by a higher volume of media in the non-infected panel (200 µL) and a lower volume in the infected panel (100 µL). Sarbecovirus- or RABV-infected microfluidic chambers were incubated at 37 °C and 5% CO_2_ for 24 h.

### Replication of SARS-CoV-2 in ferret nasal neuroepithelium

SARS-CoV-2 VIC01 infection of ferrets is described in Au et al. (2022). Briefly, ferret turbinates at 7 days post infection were fixed in 10% neutral buffered formalin (Australian Biostain). Preserved ferret turbinates were processed according to routine histological methods, embedded in paraffin wax (Leica Biosystems), and 4-µm serial sections were mounted on glass slides (Menzel Gläser). Tissue sections were deparaffinised using routine histological methods. Antigen retrieval was performed using Target Retrieval Solution, high pH (Dako) at 97°C for 30 min on a PT Link pre-treatment module (Agilent).

### Immunofluorescence staining and confocal imaging

Sarbecovirus-infected HDF51i-509 iPSC-derived neural cultures were fixed with 4% paraformaldehyde in PBS at room temperature for ≥ 1 h or overnight at 4 °C and labelled using immunocytochemistry. Fixed cells were washed twice with PBS, permeabilised with 0.1% Triton X-100 (Sigma) in PBS for 10 min, washed with PBS, and blocked with 0.5% bovine serum albumin (Sigma Bovogen Biologicals) in PBS for 30 min. Primary antibody incubation was performed overnight at 4 °C in block buffer, washed three times with PBS, and secondary antibody incubation was performed for 1 h at room temperature in block buffer. The following primary antibodies were used: 1:2000 hyperimmune horse anti-SARS polyclonal antiserum (Yu et al. 2008), 1:500 rabbit anti-SARS-CoV-2 Spike S1 (Sino Biological, cat#40,150-R007), and 1:1000 mouse anti-beta III tubulin (TuJ1), clone 2G10 (Abcam). The following secondary antibodies were used: 1:500 goat anti-horse IgG (H&L)-FITC (Abcam), 1:200 goat anti-rabbit IgG (H&L)-Alexa Fluor (AF) 568 (Invitrogen), and 1:200 goat anti-mouse IgG (H&L)-AF 647 (Invitrogen). Following secondary antibody incubation, monolayers were washed twice with PBS, then twice with deionised water before 1:4000 DAPI (Invitrogen) was added for 10 min. Monolayers were washed twice with deionised water and mounted on glass slides (Menzel-Gläser) using Vectashield (Vector Laboratories).

RABV-infected HDF51i-509 iPSC-derived neural cultures were fixed and labelled using immunocytochemistry as above with the following modification: 1:3000 rabbit anti-RABV nucleoprotein monoclonal antibody (Rahmadane et al. 2017), 1:1000 chicken anti-MAP2 (Abcam) monoclonal antibody, and 1:200 goat anti-chicken IgG (H&L)-AF 488 (Thermo Fisher) secondary antibody were used.

To assess sarbecovirus replication in ferret neuroepithelium, tissue sections were washed twice with PBS before permeabilising with 0.1% Triton X-100 in PBS for 10 min. Tissue sections were blocked with 5% bovine serum albumin and 0.1% Triton X-100 in PBS overnight at 4 °C. Primary monoclonal antibody incubation was performed overnight at 4 °C in block buffer in a humified chamber, washed three times with PBS, and secondary antibody incubation was performed for 3 h at room temperature in block buffer. The following primary antibodies were used: 1:10,000 rabbit anti-SARS-CoV-2 nucleocapsid (Sino Bio), 1:1000 mouse anti-SARS-CoV-2 nucleocapsid (Sino Bio), 1:1000 mouse anti-TuJ1, and 1:3000 rabbit anti-olfactory marker protein (OMP) [EPR19190] (Abcam). The following secondary antibodies were used: 1:200 goat anti-rabbit IgG (H&L)-Alexa Fluor (AF) 488 and 1:200 goat anti-mouse IgG (H&L)-AF 568 (Invitrogen). Following secondary antibody incubation, tissue sections were washed twice with PBS, then twice with deionised water before 1:4000 DAPI (Invitrogen) was added for 10 min. Monolayers were washed twice with deionised water and mounted on glass slides using Vectashield.

Confocal imaging was performed using a LSM 800 inverted confocal microscope (ZEISS). Images were taken as Z-stacks with or without tile scan and then maximum intensity projection was generated. Airyscan™ module was used to image ferret nasal turbinate tissues using 63 × objective with Z-stacks. All the confocal imaging and processing were performed using ZEN 2.5 Blue software (ZEISS, Oberkochen, BW, Germany).

### Quantification of infected neurons from confocal images

Confocal images of neural cultures stained with TUJ1 (neuron, red), viral antigen (green, anti-SARS2-S1; magenta, anti-SARS), and DAPI (blue, nucleus) were taken with 20 × objective covering 100–400 neural cells in a single image. Total number of cells (DAPI stained nuclei) were counted using ImageJ particle analyser plugin and watershed function, with the following parameters: size (inch^2^) 0.005–infinity and circularity 0.00–1.00. The number of TUJ1 and viral antigen–positive neurons were counted manually in the same image to determine the % of infected neurons.

### Cell-associated sarbecovirus RNA quantitation

RNA was extracted from sarbecovirus-infected neural cultures using MagMAX Viral RNA Isolation Kit (Applied Biosystems) using a KingFisher magnetic particle processor (Thermo Fisher Scientific). cDNA was synthesised using SuperScript IV VILO Master Mix with ezDNase enzyme (Thermo Fisher Scientific) according to manufacturers’ instructions.

Cell-associated sarbecovirus RNA quantitation was performed in duplicate by quantitative real-time PCR using AgPath-ID One-Step RT-PCR kit (Applied Biosystems), sarbeco E gene reverse primer and sarbeco hydrolysis probe (Corman et al. 2020) with the 3′ quencher modified to MGB, and a modified E gene forward primer (Marsh et al. 2021) on a QuantStudio 6 Flex Real-Time PCR System (Thermo Fisher Scientific) according to manufacturers’ instructions. Sarbecovirus E gene copy numbers were calculated using cycle threshold (*C*_T_) and a standard curve of known copy number.

### Statistical analysis

Statistical analyses (ordinary one-way ANOVA with Tukey’s multiple comparison) were performed using GraphPad Prism, version 9.3.1 (GraphPad Software). Data are presented as mean ± SEM. *p < 0.05, **p < 0.01, ****p < 0.0001.

## Results

### Sarbecoviruses display limited ability to infect human iPSC-derived neural culture

**Fig. 1:**
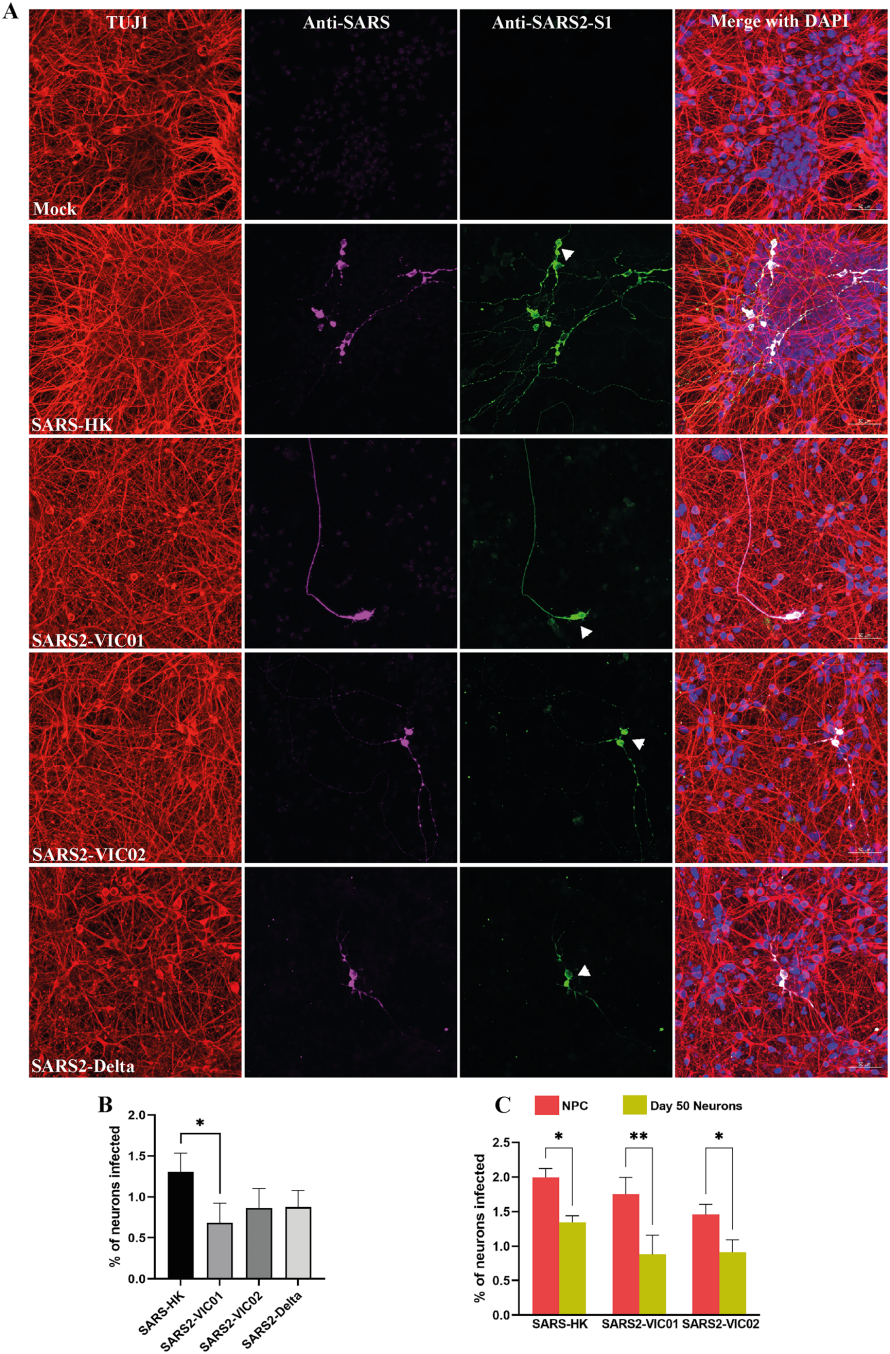
Infection of iPSC-derived human neural cultures with sarbecoviruses. A Representative confocal images of stem cell–derived human neural cultures at differentiation day 21–24 infected with different strains of SARS and SARS-CoV-2 at MOI 1 for 24 h. Neurons are identified by staining with TUJ1 (red) and viral antigen identified by staining with anti-SARS S1 antibody (green) and horse antiserum recognising SARS coronavirus (magenta). B Quantification of sarbecovirus-infected day 21–24 differentiated neurons at 24 hpi. C Quantification of sarbecovirus-infected undifferentiated NPCs and day 50 differentiated neurons at 24 hpi. 100–400 neural cells in a single image, totalling at least 1000 neurons, were counted using ImageJ from one to three independent experiments (*n* = 1, Delta; *n* = 3, SARS-HK, VIC01, VIC02). Error bars represent mean ± SEM. *p < 0.05, **p < 0.001

To determine whether sarbecoviruses productively infect CNS-origin neural cells, we generated human neural cultures consisting of neurons and astrocytes from an iPSC line (HDF51i-509), after initial derivation and expansion into undifferentiated NPCs (Sundaramoorthy et al. 2020a). Undifferentiated NPCs as well as differentiated human neural cultures for medium term (21–24 days) or long term (50 days) were infected with different strains of sarbecoviruses including SARS (Hong Kong strain) and SARS-CoV-2 (ancestral VIC01 and VIC02 strains, and delta strain) for up to 72 h (Fig. 1, Additional file 1, Additional file 2, Additional file 3). SARS and SARS-CoV-2 infected only a minor proportion of neurons (< 2%) at 24-h post infection (hpi) in 21–24-day-old neural cultures (Fig. 1A). Viral antigen was localised in both the cell body and in the neurites (axons and dendrites, Fig. 1A). Cell–cell fusion of infected neurons was observed, which aligned with previous studies examining fusogenic capacity of SARS-CoV-2 (Zhang et al. 2020; Zeng et al. 2022); however, this did not lead to the formation of virus-induced syncytia (Fig. 1A, arrows). With the exception of cell–cell fusion, we did not observe cytopathology in infected neurons. Quantification of neuron-associated viral antigen in confocal images at 24 hpi showed slightly higher percentage of infection with the SARS-HK strain compared to the SARS-CoV-2 strains (Fig. 1B). However, the percentage of infection at 24 hpi among all the strains studied was < 2%. To determine whether sarbecovirus infection of neural cultures was productive, we analysed the course of infection with SARS (SARS-HK) and SARS-CoV-2 (VIC01, VIC02, and Delta; Additional file 1) in day 21–24 neural cultures for up to 72 hpi. The rate of neuronal infection at 72 h was similar to that observed at 24 hpi (Additional file 1), indicating that the infection did not appear to increase overtime and that sarbecoviruses displayed limited ability to infect iPSC-derived neurons.

While neurons are postmitotic, neural progenitor cell populations are capable of proliferating which could be more susceptible to sarbecovirus infection and act as sites of viral replication in human brain. Hence, we examined whether neuronal infection in our model could vary with the stage of neural cell differentiation from iPSC-derived NPCs. Undifferentiated NPCs and neural cultures that were differentiated for up to 50 days were infected with SARS (HK strain) and SARS-CoV-2 (VIC01 and VIC02 strains) for 24 and 72 hpi. Day 50 differentiated neurons showed similar levels of infection to day 21–24 differentiated neurons (Additional file 2A), but the undifferentiated NPC showed slightly higher levels of infection (Additional file 3A, Fig. 1C). This indicated that these SARS and SARS-CoV-2 strains may have reduced potential to infect cells as they mature into neurons. However, as per results for day 21–24 differentiated neurons, viral infection did not increase at 72 h in NPCs or day 50 infected neural cultures (Additional file 2B; Additional file 3B). We then examined the levels of cell-associated viral RNA in infected neural cells by qPCR (Additional file 4A) and virus titre in infected neural cultures (Additional file 4B). While viral RNA was detected in infected neural cultures and there was a low titre of infectious virus in the culture media, this did not increase overtime at 48 and 72 h. Rather, a gradual degradation of viral titres was observed at 48 and 72 h (Additional file 4B). These results were consistent with confocal imaging and together indicates a lack of productive viral replication in neural cultures infected with both the SARS and SARS-CoV-2 strains.

Collectively, we observed infection of a minor proportion of neurons by sarbecoviruses without productive replication, consistent with other studies (Bauer et al. 2021, 2022b). Furthermore, this low infectivity of sarbecoviruses in forebrain neurons decreased with the maturity of the neural cultures.

### Microfluidic-derived ex vivo model of interconnected human neurons shows inability of sarbecoviruses to transmit between neurons

**Fig. 2:**
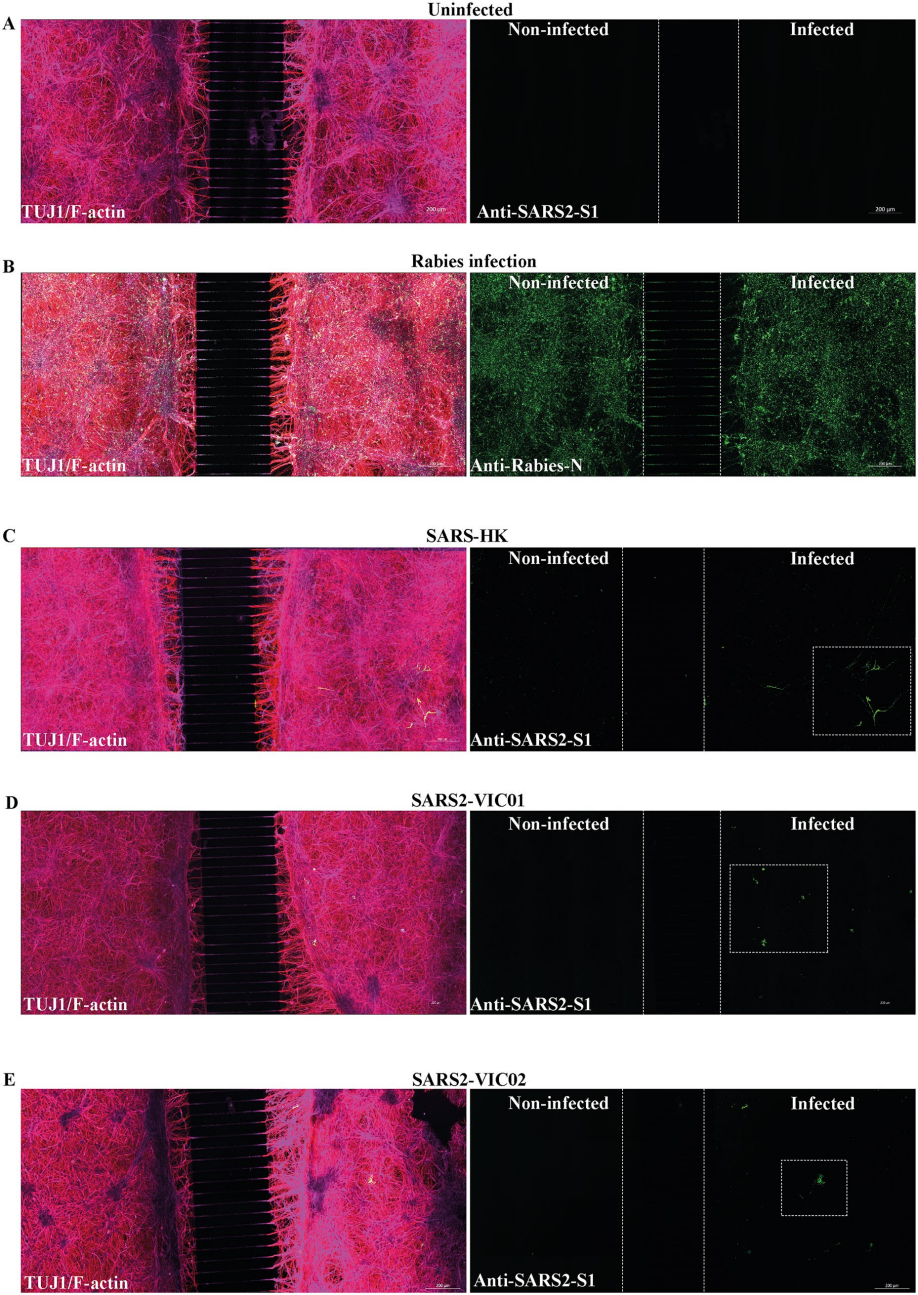
Sarbecovirus infection of ex vivo model of interconnected human neural network to assess trans-synaptic transmission. **A** Stem cell–derived NPCs were seeded in both panels of microfluidic device separated by microchannels which allows only the neurites to pass through. After seeding, NPCs were differentiated for 20 (RABV) to 21 days (sarbecoviruses) to generate neurons in both panels interconnected by neurites through the microchannels. The neuronal network is identified by TUJ1 staining (red) and F-actin stain to visualise the neural architecture (magenta). The ex vivo models were infected MOI 1 with RABV for 48 h, which served as a positive control for trans-synaptic transmission (**B**) or different strains of sarbecoviruses (**C, D, E**) at for 24 h. To prevent passive diffusion of virus from the infected panel into the non-infected panel, a higher volume of media is maintained in the non-infected panel. After 24 h of infection, neural cultures were fixed, stained, and imaged in the microfluidic device. Tile images were taken with 20 × objective with Z-stacks and stitched together. Image **B** shows trans-synaptic spread of RABV from the infected to non-infected panel identified by staining with a rabies anti-nucleoprotein antibody (green). Images **C, D,** and **E** show SARS and SARS-CoV-2 infections stained with anti-SARS2-S1 antibody (green) in a subset of neurons in the infected panel (boxed area) but viral antigen was not observed in the non-infected panel

To date, it remains unclear whether sarbecoviruses are capable of transmitting within neurites (axons and dendrites) to neighbouring connected neurons. This would allow the trans-synaptic spread of sarbecoviruses within a subset of human neurons without active release outside the cell. To examine this, we used an ex vivo model of interconnected human neuronal network using microfluidics. In this previously described model (Sundaramoorthy et al. 2020a, b), two populations of forebrain neural cultures are differentiated from NPCs in panels on either side of microchannels. Upon differentiation, the neurons develop neurites which grow through the microchannels and connect the two neuronal populations forming a network mimicking the circuits in human brain (Fig. 2A).

We confirmed the synaptic connectivity between the two neuronal populations in our microfluidic model using RABV (Fig. 2B). RABV is known to exclusively transfer via synaptic connections between neurons and hence widely used as a trans-synaptic neural tracer (Suzuki et al. 2020). The maintenance of unidirectional flow of culture media from the uninfected to infected panel also prevents diffusion of virus between the two panels. When RABV was added to the infected panel, we observed efficient viral spread to the non-infected panel within 24 h by trans-synaptic transmission (Fig. 2B). This demonstrated the presence of functional connectivity between human neurons in our model. We then infected this model with different strains of sarbecoviruses (SARS-HK, SARS2-VIC01, and VIC02) for 24 h. In these infections (Fig. 2C–E), we observed the infection of a subset of neurons in the infected panel similar to our previous observation (Fig. 1). However, while the viral antigen was observed in the neurites of infected neurons (Fig. 2C–E, boxed sections), this did not result in the trans-synaptic spread of infection from the infected to non-infected microfluid panel. This suggests that the both SARS and SARS-CoV-2 strains lack the inherent ability to transfer between human neurons by trans-synaptic transmission.

### Absence of neuronal infection in the olfactory epithelium of SARS-CoV-2-infected ferrets

**Fig. 3:**
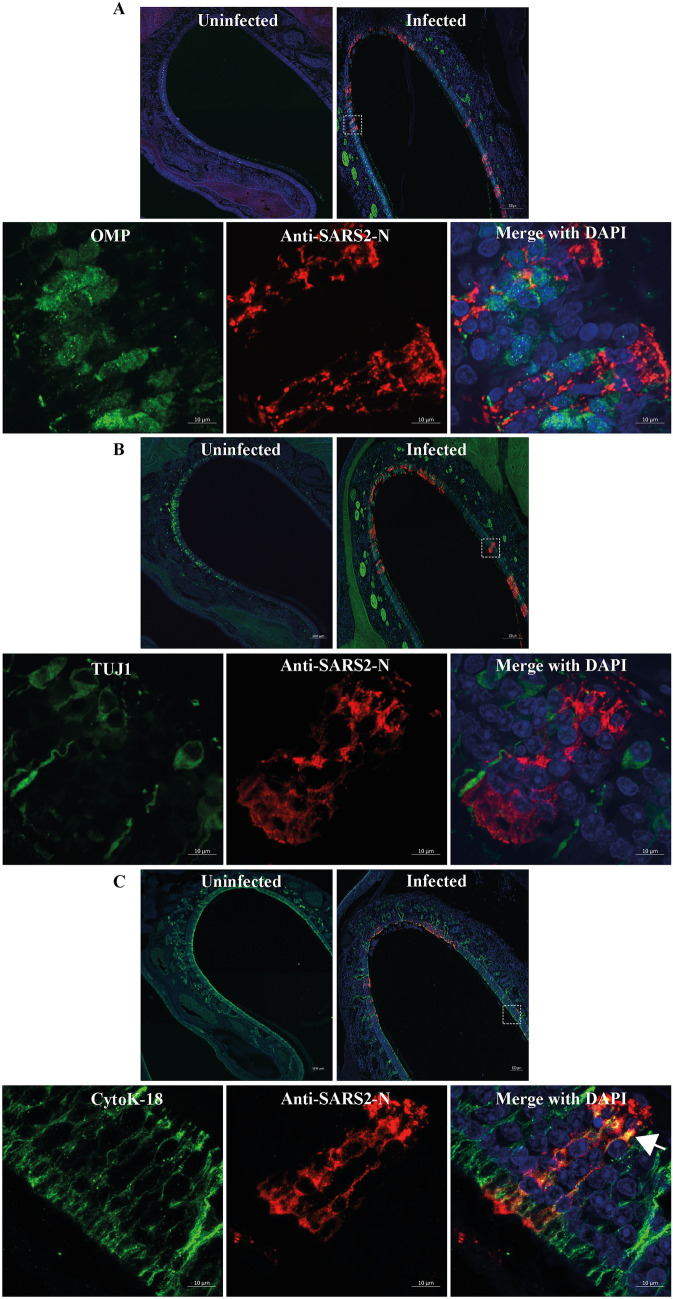
Detection of SARS-CoV-2 antigen in ferret nasal neuroepithelium. High-resolution Airyscan confocal imaging of ferret nasal olfactory epithelium infected with SARS-CoV-2 (VIC01 strain). Ferrets were inoculated with 4.64 × 10^4^ TCID_50_ VIC01 via the intranasal route and ferret tissue collected 7 dpi (Au et al. 2022). Ferret tissue sections analysed herein were stained with anti-SARS-CoV-2 nucleocapsid antibody (red) (**A–C**), mature olfactory neuronal marker (OMP) (**A**), immature olfactory neuronal marker (TUJ1) (**B**), or epithelial (sustentacular) cell marker (cytoK-18) (**C**). The top two panels of images **A–C** are images taken with 20 × objective, while the bottom three panels are images taken with 63 × objective using a high-resolution Airyscan module. All images were taken as z-stacks and the maximum intensity projection of z-stacks is shown. Images show that the viral antigen (green) is more prominently co-localised with the sustentacular cells (cytoK-18, red), indicated by white arrows. While virus antigen is observed close to the OMP or TUJ1 positive neurons in images **A** and **B**, a clear localisation within the neurons is not observed compared to sustentacular cells (**C**)

We then examined whether the low potential for SARS-CoV-2 to infect stem cell–derived neurons also applies to olfactory neurons in an in vivo model of sarbecovirus infection. We used the olfactory epithelium tissue of SARS-CoV-2-infected ferrets described previously (Au et al. 2022) and performed high-resolution confocal imaging to examine the infection in olfactory neurons (Fig. 3). The ferrets infected in this previous study showed sparingly distributed viral antigen in respiratory and olfactory epithelium at 5–7 days post infection (Au et al. 2022). We used OMP (Fig. 3A) and TUJ1 (Fig. 3B) as markers for mature and immature olfactory neurons, respectively, as well as cytoK-18 as a marker of sustentacular cells (Fig. 3C) which wrap around the olfactory neurons. Our confocal imaging showed patches of viral antigen positivity distributed in the olfactory epithelium (Fig. 3, Additional file 5). While low-magnification imaging indicated the viral antigen could be present in olfactory neurons, high-resolution confocal imaging indicated that this viral antigen is present within the sustentacular cells surrounding the neurons (Fig. 3; Additional file 5, arrows, Additional file 6). We did not observe any infection of TUJI (Additional file 7) or OMP positive neurons in the olfactory epithelium (Fig. 3, Additional file 5). This suggests that sarbecovirus infection in the ferret nasal olfactory neuroepithelium is largely restricted to sustentacular cells with no evidence for infection of olfactory neurons despite their close proximity. Collectively, these results correlate with the limited potential for SARS-CoV-2 to infect neurons, as observed in our human ex vivo neural model.

## Discussion

It has become clear that SARS-CoV-2, despite being a primarily respiratory pathogen, affects multiple tissues and organs including the nervous system. Many studies have now explored the neurotropism and neuropathogenic potential of SARS-CoV-2, sometimes with contradicting results (reviewed in Bauer et al. (2022a)). One important hurdle in understanding the neuropathogenesis of sarbecoviruses is the complexity of human brain with multiple cell types and neuronal subtypes. Hence, it is difficult to model and investigate the tropism and progress of viral infection in different parts of the nervous system. Investigation in post-mortem tissues could also be affected by the stages of infection and individual patient heterogeneity. But increasing evidence suggests that neuropathogenesis of sarbecoviruses is likely to be complex and not solely caused by direct neuronal infection. Neurological complications in SARS-CoV-2 could be caused by indirect non-neuronal mechanisms such as immune mediated neuroinflammation including microglial overactivation (Albornoz et al. 2022), astrocyte-mediated neuropathology (Andrews et al. 2022; Huang and Fishell 2022), or neurovascular thrombosis (Spudich and Nath 2022) due to hypercoagulable state during SARS-CoV-2 infection.

In this study, we demonstrated a low potential for SARS and SARS-CoV-2 to infect and replicate in stem cell–derived human neurons. We examined sarbecovirus infection of stem cell–derived neurons at different stages of differentiation which indicated that both SARS and SARS-CoV-2 show lower potential to infect mature neurons compared to NPCs. Our data suggests that in iPSC-derived forebrain-type neuron enriched cultures (Sundaramoorthy et al. 2020a), there is not productive viral replication despite the infection of few neurons. Our data showing low neuronal infection is consistent with earlier published studies using similar stem cell model systems (Bauer et al. 2021, 2022b). However, these findings contradict previous studies using NPCs or more complex 3D organoid model systems containing multiple cell types (Zhang et al. 2020; Ramani et al. 2020). In other studies, utilising 3D organoid models (Kong et al. 2022; Jacob et al. 2020; Pellegrini et al. 2020), infection was also primarily observed in non-neuronal cells which could contribute to neuronal infection or neuropathology. Further investigation is required to understand the role of non-neuronal cell types in different parts of the nervous system in the tropism and replication of sarbecoviruses.

A widely suggested mechanism of SARS-CoV-2 neuroinvasion is entry from the upper respiratory tract where there is high viral load, into the olfactory bulb and then the brain via olfactory nerves or other cranial nerves. This route of entry from the nose into the CNS has been described for other clinically important respiratory pathogens such as influenza A virus (reviewed in van Riel et al. (2015)). A potential mechanism for viral entry into the CNS is axonal trafficking within cranial nerves and trans-synaptic transmission of SARS-CoV-2 to neurons of the olfactory bulb and then transport into the brain. However, this requires mechanisms to travel within the nerves using specific axonal trafficking mechanisms and transmit across synapses. In this study, we examined for the first time whether sarbecoviruses could exhibit such a mechanism. Using a microfluidic derived model of interconnected human forebrain-type neurons, we demonstrated that both SARS and SARS-CoV-2 viruses do not have the ability to transmit within axons. While we do acknowledge the limitation of our model system consisting of only forebrain-type neurons, we show that sarbecoviruses lack an inbuilt ability to hijack and repurpose the axonal trafficking machinery that is largely conserved across neuronal subtypes from nose to brain (Beier et al. 2011). This suggests both these viruses do not have the ability to utilise axonal trafficking mechanisms to enter the brain from the nose. However, other possible ways of virus transfer along the cranial nerve such as passive diffusion between olfactory ensheathing cells needs to be examined (van Riel et al. 2015). Alternatively, the virus may also enter the brain by hematogenous spread through the blood brain barrier (reviewed in Bauer et al. (2022a)). Interestingly, the S1 protein of SARS-CoV-2 has also been shown to have an ability to transfer across the blood brain barrier in the mouse model (Rhea et al. 2021).

## Conclusions

In summary, this study adds further evidence towards understanding the mechanisms behind neurotropism and neuropathology of sarbecoviruses. Our data suggests that invasion of SARS and SARS-CoV-2 into the brain is likely mediated by mechanisms other than axonal trafficking. Additionally, we demonstrated low potential for sarbecovirus infection of human iPSC–derived neurons. Together, our data warrants investigation of mechanisms of neuropathogenesis that are alternative to direct neuronal targeting and intraneuronal transmission in sarbecovirus infections.

## Supplementary information

**Additional file 1. Sarbecovirus infection of iPSC-derived human neural cultures at differentiation day 21–24. A)** Infection of stem cell-derived human neural cultures at differentiation day 21–24 with different strains of sarbecoviruses at MOI 1 for 72 h. Neurons are stained with TUJ1 (red) and viral antigen were stained with anti-SARS S1 antibody (green) and horse antiserum recognising SARS coronavirus (magenta). **B)** Quantification of sarbecovirus-infected neurons after 72 h infection at MOI 1 in 21–24 days differentiated neural cultures. 100–400 neural cells in a single image, totalling at least 1000 neurons, were counted using imageJ from 1–3 independent experiments (n = 1, Delta; n = 3, SARS-HK, VIC01, VIC02). Error bars represent mean ± SEM. ns, not significant.

**Additional file 2. Sarbecovirus infection of iPSC-derived human neural cultures at differentiation day 50. A)** Infection of stem cell-derived human neurons at differentiation day 50 with different strains of coronaviruses at MOI 1 for 24 or 72 h. Neurons are marked by TUJI (red) staining and virus antigen with anti-SARS S1 antibody (green) and horse anti-SARS coronavirus staining (magenta). **B)** Quantification of infected neurons after 24 h or 72 h infection at MOI 1 in 50 days differentiated neural cultures. 100–400 neural cells in a single image, totalling at least 1000 neurons, were counted using imageJ from 3 independent experiments. Error bars represent mean ± SEM. ns, not significant.

**Additional file 3. Sarbecovirus infection of iPSCs-derived undifferentiated NPCs. A)** Infection of undifferentiated neuronal precursor cells (NPCs) with different strains of coronaviruses at MOI 1 for 24 or 72 h. Immature neurons are identifed by TUJ1 (red) staining and viral antigen identified using anti-SARS S1 antibody (green) and horse anti-SARS coronavirus antiserum (magenta). **B)** Quantification of infected NPCs after 24 h or 72 h infection at MOI 1. 100–400 neural cells in a single image, totalling at least 1000 neurons, were counted using imageJ from 3 independent experiments. Error bars represent mean ± SEM. ns, not significant.

**Additional file 4. Replication of sarbecoviruses in human neural cultures.** To assess susceptibility of neural cultures to sarbecovirus infection and examine replication dynamics, cell associated viral genome (**A**) or infectious virus titre in tissue culture supernatant (**B**) were quantitated. **A)** RT-qPCR to determine cell-associated sarbecovirus genome in stem cell-derived neural cultures (21–24 days differentiation) infected with sarbecoviruses at MOI 1 for 24, 48 and 72 h. **B)** Virus titres as determined by TCID_50_ assay of neural cultures infected with coronaviruses at MOI 1 for 24, 48 and 72 h. Individual data points shown. Quantitation of cell associated viral genome and virus titres were performed on 1 independent experiment. Dotted line indicates limit of assay detection.

**Additional file 5. Detection of SARS-CoV-2 antigen in ferret nasal neuroepithelium.** Additional representative confocal images of ferret nasal olfactory epithelium infected with SARS-CoV-2 (VIC01 strain). Anti-SARS-CoV-2 nucleocapsid antibody is used to detect viral antigen (red) with TUJ1, OMP (olfactory neurons) or cytoK-18 (sustentacular cells). Representative images shown from two regions in the same tissue for each stain. High-resolution confocal images were taken using 63 × objective with airy-scan module. All images were taken as z-stacks and the maximum intensity projection of z-stacks is shown. Images show co-localisation of viral antigen with sustentacular cells but not with olfactory neurons.

**Additional file 6. Z-stack visualisation of SARS-CoV-2 antigen in ferret nasal neuroepithelium.** Representative videos showing the Z-stack series of confocal images of SARS-CoV-2 (VIC01 strain) infected ferret nasal olfactory epithelium stained with anti-SARS-CoV-2 nucleocapsid antibody to detect viral antigen (red) and sustentacular cell marker cytokeratin 18 (green).

**Additional file 7. Z-stack visualisation of SARS-CoV-2 antigen in ferret nasal neuroepithelium.** Representative videos showing the Z-stack series of confocal images of SARS-CoV-2 (VIC01 strain) infected ferret nasal olfactory epithelium stained with anti-SARS-CoV-2 nucleocapsid antibody to detect viral antigen (red) and neuronal marker TUJ1 (green).

### Supplementary Information

Below is the link to the electronic supplementary material.

Supplementary file1 (TIF 30485 KB)

Supplementary file2 (TIF 20282 KB)

Supplementary file3 (TIF 19600 KB)

Supplementary file4 (TIF 4583 KB)

Supplementary file5 (TIF 22693 KB)

Supplementary file6 (AVI 21811 KB)

Supplementary file7 (AVI 19732 KB)

## Data Availability

The datasets used and/or analysed during the current study are available from the corresponding author on reasonable request.

## Abbreviations

CNS: Central nervous system
hpi: Hours post infection
iPSCs: Induced pluripotent stem cells
MOI: Multiplicity of infection
NPC: Neuronal precursor cell
OMP: Olfactory marker protein
PBS: Phosphate-buffered saline
RABV: Rabies virus
SARS: Severe acute respiratory syndrome coronavirus
SARS-CoV-2: Severe acute respiratory syndrome coronavirus 2
TuJ1: Beta III tubulin
