# Supplementary figures and images for: SARS-CoV and SARS-CoV-2 display limited neuronal infection and lack the ability to transmit within synaptically connected axons in stem cell–derived human neurons
Source: J Neurovirol. 2024 Jan 3;30(1):39–51. doi: 10.1007/s13365-023-01187-3 (PMC11035468; doi:10.1007/s13365-023-01187-3)

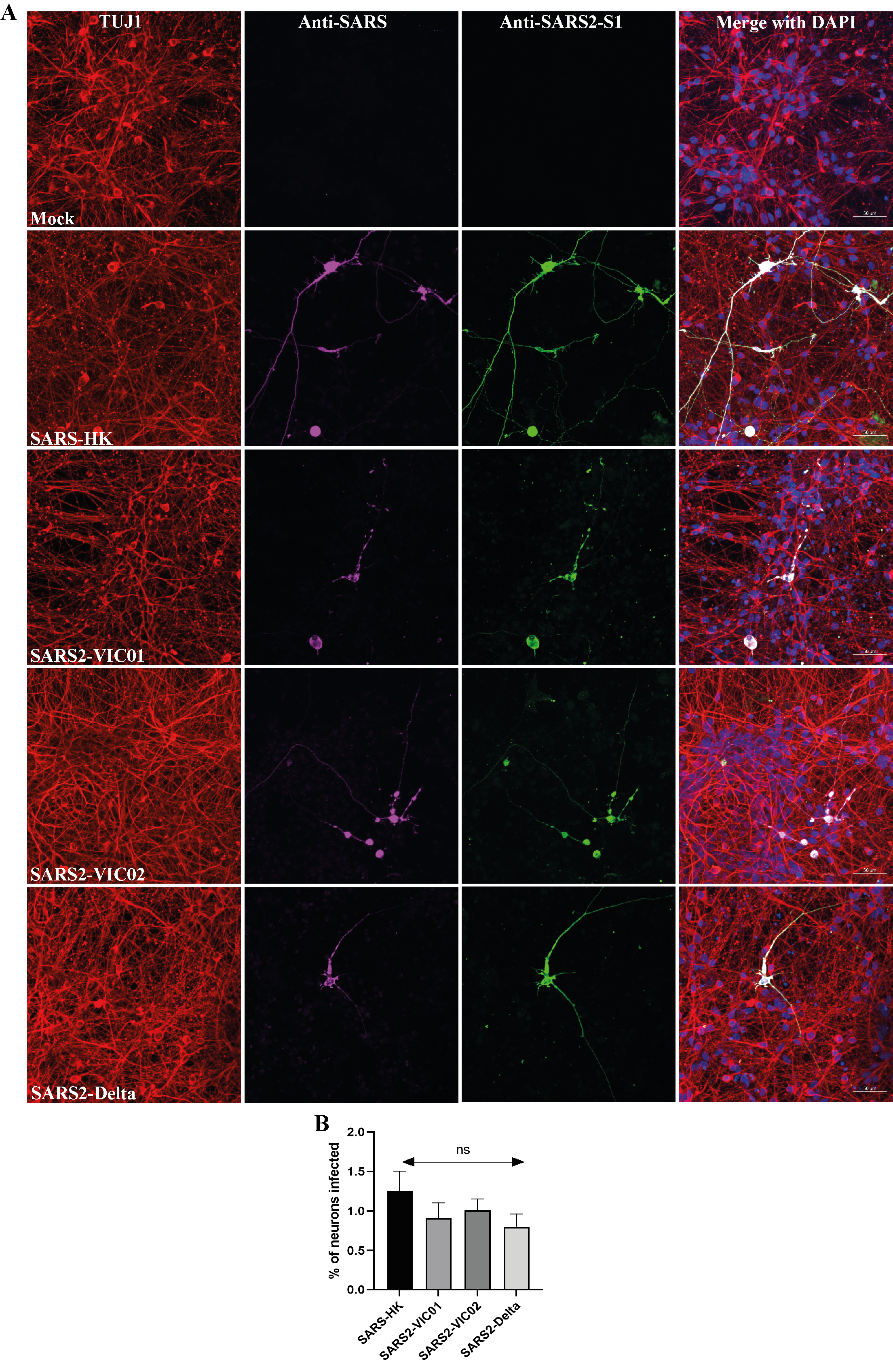

Supplement: Supplementary file 1 — Supplementary file1 (TIF 30485 KB) [file 13365_2023_1187_MOESM1_ESM.tif]

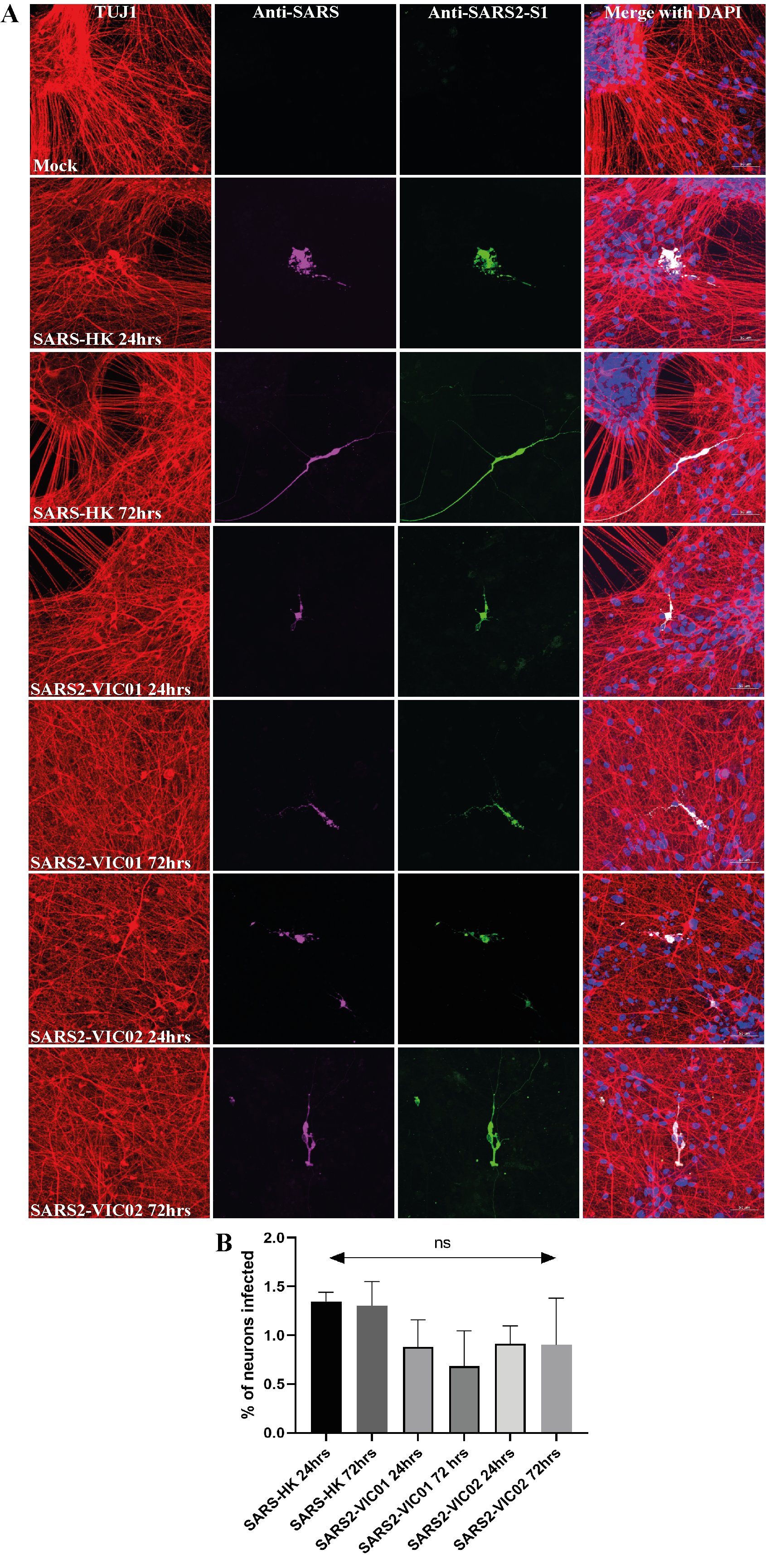

Supplement: Supplementary file 2 — Supplementary file2 (TIF 20282 KB) [file 13365_2023_1187_MOESM2_ESM.tif]

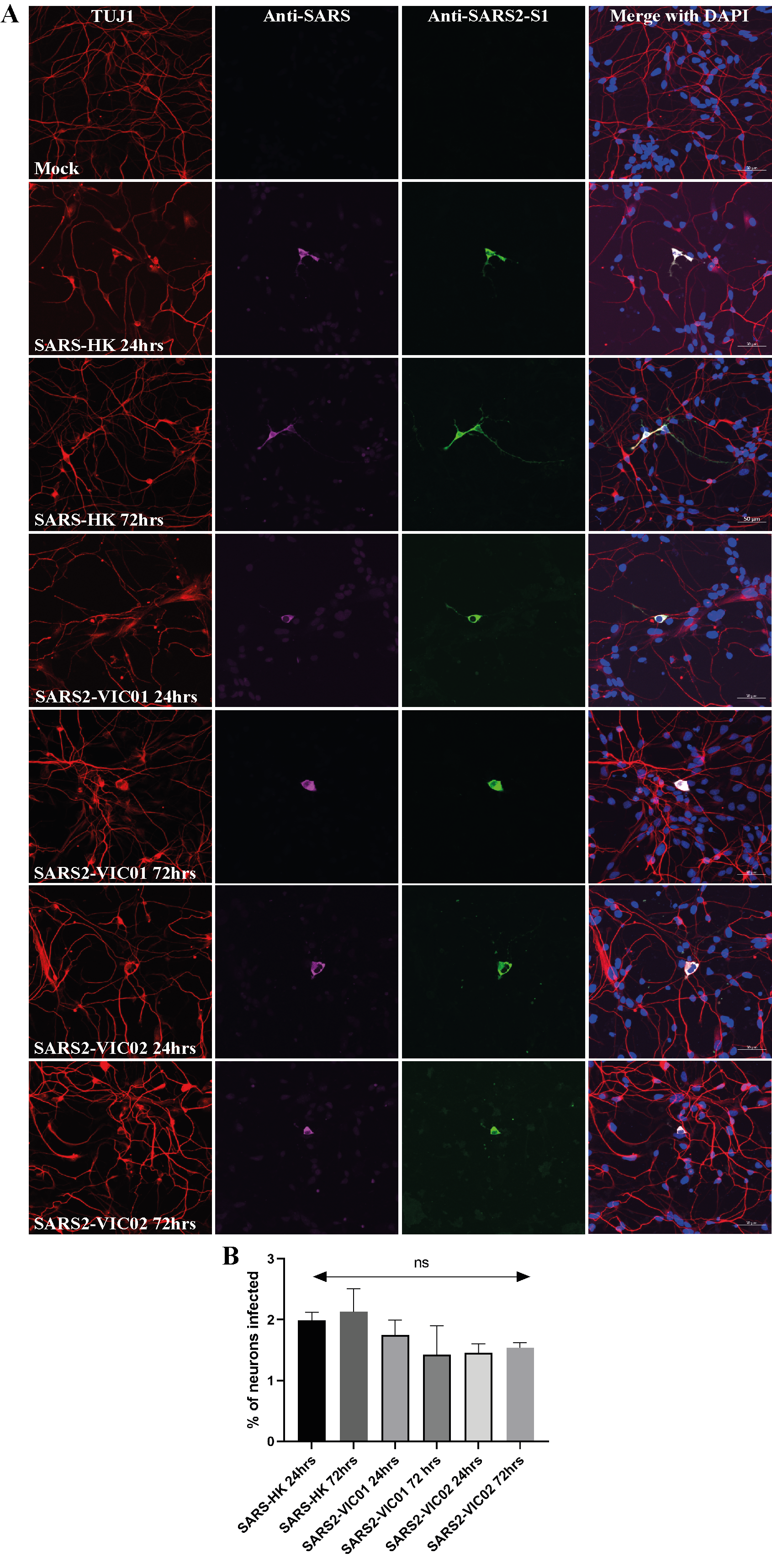

Supplement: Supplementary file 3 — Supplementary file3 (TIF 19600 KB) [file 13365_2023_1187_MOESM3_ESM.tif]

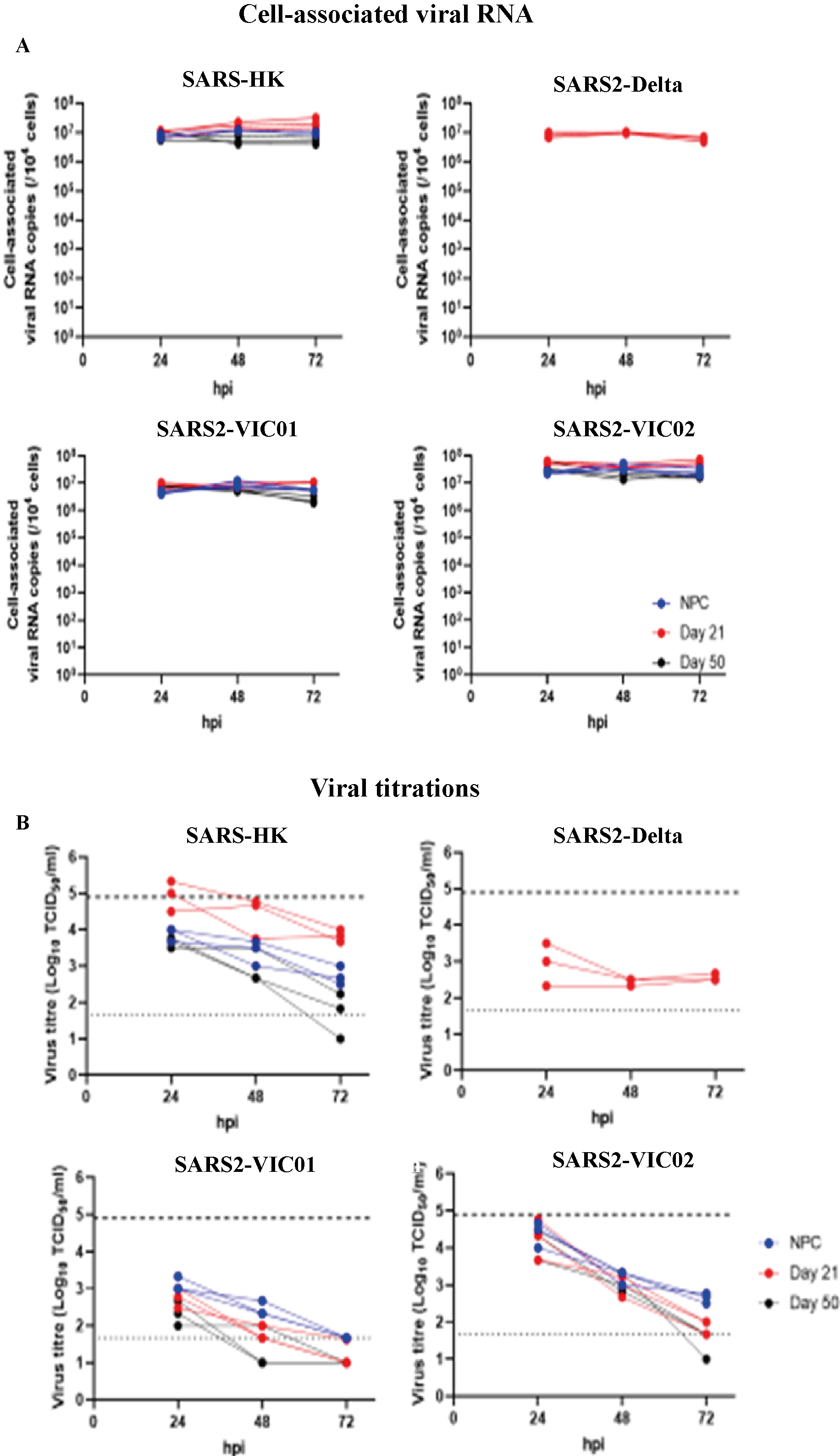

Supplement: Supplementary file 4 — Supplementary file4 (TIF 4583 KB) [file 13365_2023_1187_MOESM4_ESM.tif]

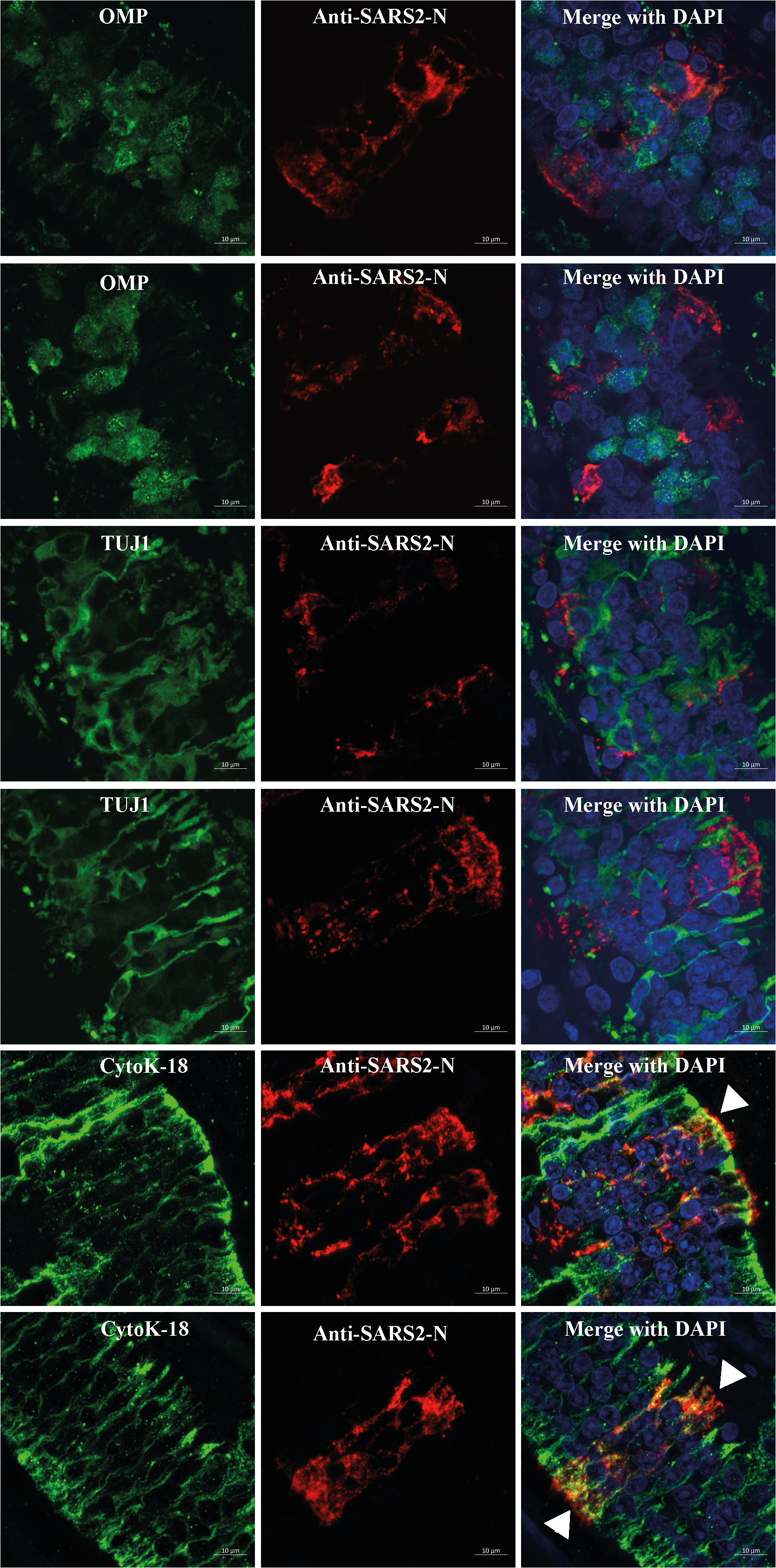

Supplement: Supplementary file 5 — Supplementary file5 (TIF 22693 KB) [file 13365_2023_1187_MOESM5_ESM.tif]
